# Murine glial progenitor cells transplantation and synthetic PreImplantation Factor (sPIF) reduces inflammation and early motor impairment in ALS mice

**DOI:** 10.1038/s41598-022-08064-9

**Published:** 2022-03-07

**Authors:** Karolina A. Ambrożkiewicz, Urszula Kozłowska, Valerie Haesler, Eytan R. Barnea, Martin Mueller, Maciej Kurpisz

**Affiliations:** 1 Institute of Human Genetics, Polish Academy of Sciences, Poznan, Poland; 2 Institute of Bioorganic Chemistry, Polish Academy of Sciences, Poznan, Poland; 3 Department of Obstetrics and Gynecology, Department of Biomedical Research, University Hospital Bern, University of Bern, Bern, Switzerland; 4 The Society for the Investigation of Early Pregnancy (SIEP), Cherry Hill, NJ USA; 5 BioIncept LLC, Cherry Hill, NJ USA

**Keywords:** Immunology, Stem cells, Neurodegenerative diseases

## Abstract

Amyotrophic lateral sclerosis (ALS) is a progressive motor neuronal disorder characterized by neuronal degeneration and currently no effective cure is available to stop or delay the disease from progression. Transplantation of murine glial-restricted precursors (mGRPs) is an attractive strategy to modulate ALS development and advancements such as the use of immune modulators could potentially extend graft survival and function. Using a well-established ALS transgenic mouse model (SOD1^G93A^), we tested mGRPs in combination with the immune modulators synthetic PreImplantation Factor (sPIF), Tacrolimus (Tac), and Costimulatory Blockade (CB). We report that transplantation of mGRPs into the cisterna magna did not result in increased mice survival. The addition of immunomodulatory regimes again did not increase mice lifespan but improved motor functions and sPIF was superior compared to other immune modulators. Immune modulators did not affect mGRPs engraftment significantly but reduced pro-inflammatory cytokine production. Finally, sPIF and CB reduced the number of microglial cells and prevented neuronal number loss. Given the safety profile and a neuroprotective potential of sPIF, we envision its clinical application in near future.

## Introduction

Amyotrophic lateral sclerosis (ALS) is a progressive motor neuronal disorder characterized by degeneration of the upper and lower motor neurons of the cortex, the brain stem, and the spinal cord. Although the start is focal, the heterogeneous manifestations and disseminating patterns suggest the spreading among advancement motor neuronal pools with glial partners^1^. The end stage of ALS is the respiratory failure (about 3–4 years after the onset) and currently no effective cure is available to stop or delay the disease from progression. Therefore, testing of new therapeutic approaches is essential and one well-defined animal model is the SOD1^G93A^ transgenic mouse model^2^. In this model, mice express a G93A mutant form of human SOD1 and develop progressive loss of upper and lower motor neurons. Typically, within 4–5 month of symptom onset mice express muscle denervation, weakness, and atrophy resulting in subsequent death^3^.

The importance of glial cells in the degeneration of motor neurons emerged after synthesis of SOD1 mutants and consecutive selective silencing^1^. The activation of innate immune cells such as microglia and excessive extracellular production of superoxide contribute substantially to ALS development and progression. Not surprisingly, the transplantation of murine glial-restricted precursors (mGRPs) is an attractive strategy to modulate ALS development^4^. Notably, GRPs are oligodendrocytic and astrocytic cell lineage precursors and recent advancements support its clinical application for ALS treatment^5^. However, the optimal therapeutic window, the ideal cell type and dose, and delivery site or method are still non optimized. The recently updated Cochrane review underlines the need of further research to explore combination of cellular therapy and novel therapeutics^6^. Such adjuvant therapeutics may include synthetic PreImplantation Factor (sPIF), Tacrolimus (Tac), and Costimulatory Blockade (CB).

PIF is a small 15-amino acid pregnancy derived peptide, which is secreted by the trophoblast/embryo^7,8^. Besides immunomodulatory properties in and outside pregnancy^7,9^, a synthetic version of PIF (PIF analog: sPIF) was successfully tested in animal models of multiple immune disorders^10–15^ and received a Fast-Track FDA approval (autoimmune diseases of non-pregnant subjects clinicaltrials.gov, NCT02239562). sPIF was able to reverse and prevent paralysis and restore myelination through inhibiting neuro-inflammation in murine models of experimental autoimmune encephalomyelitis^16^. The neuroprotective property of sPIF was further underscored by its ability to mitigate neuronal loss and microglial activation in murine model of immature brain injury as well^9,12,13,17^. Tac is a calcineurin inhibitor and a first-line immunosuppressive drug used after organ transplant^18^, which additionally may enhance neuronal regeneration^19^. Finally, the T cells feature crucial immune response toward allografts and co-stimulatory molecules involved in T-cell activation may be targeted as well^20^. The so called costimulatory blockade (CB) includes CTLA4 (Cytotoxic T-lymphocyte-associated antigen 4) and MR-1 (anti-CD154 antibody/CD40L monoclonal antibody) targeting CD40/CD154 axis^21,22^.

Together, immune-modulatory therapeutics in combination with stem cell transplantation possess the potential to enhanced cell protection/regeneration in ALS. We aimed to evaluate the potential of sPIF, Tac and CB in combination with mGRPs transplantation using the SOD1^G93A^ transgenic mouse model.

## Results

### mGRPs and sPIF improve motor function but not survival

**Figure 1:**
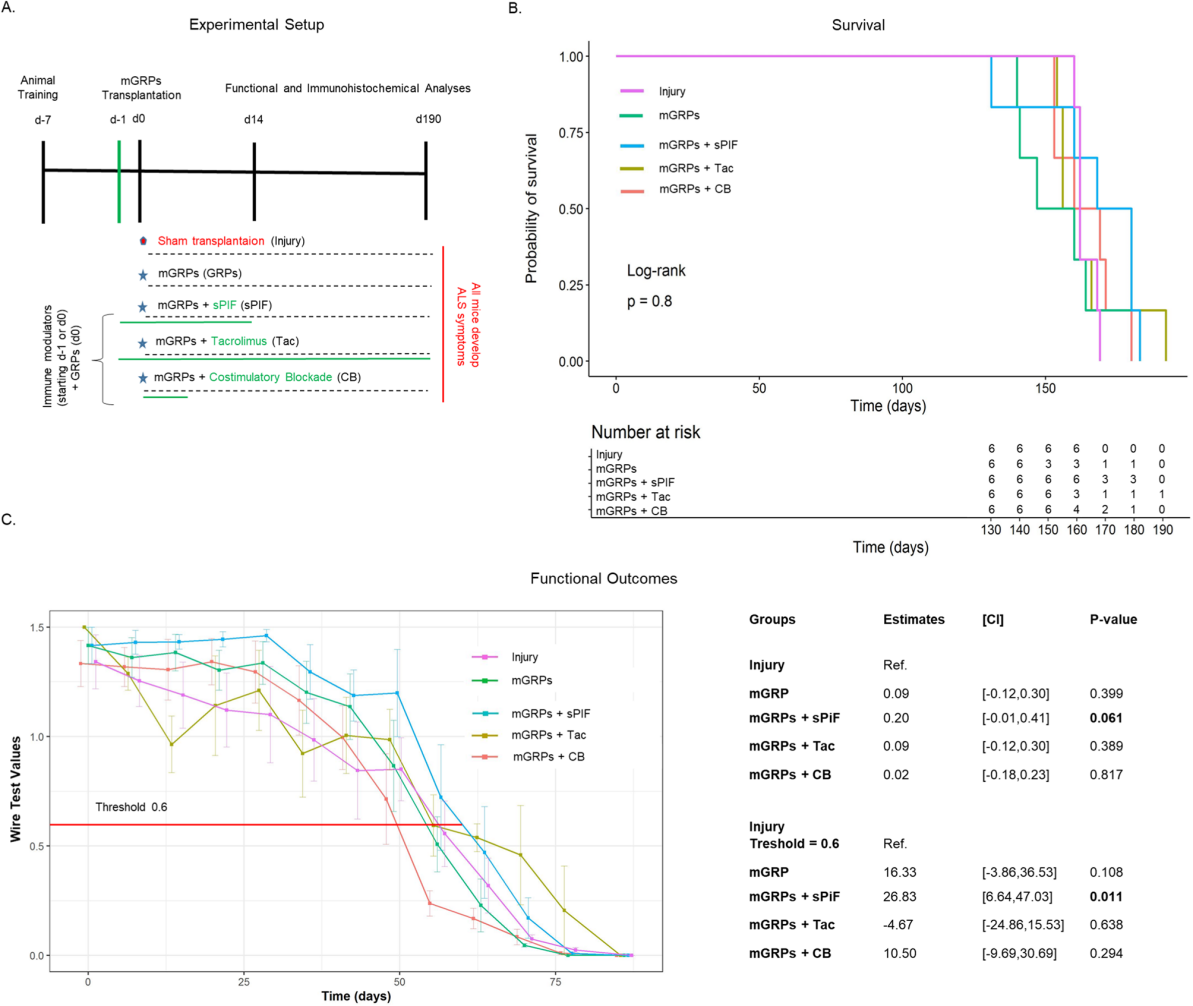
Experimental setup and clinical outcomes. (**A**) Cartoon summarizing the experimental setup and treatment groups in the ALS mouse model (n = 6 each group). The timing and duration of immunomodulatory drug applications are shown in green and GRPs transplantation in red. All animals received GRPs except Injury group, which received a sham transplantation. (**B**) Kaplan–Meier curves indicating survival by group. sPIF transplantation seems superior but did not reach statistical significance. (**C**) Functional assessments using hanging wire test by group. sPIF treatment resulted in significant improvement of motor outcome. mGRPs: glial-restricted precursors; sPIF: synthetic PreImplantation Factor; Tac: Tacrolimus, CB: co-stimulatory blockade. Differences between groups were analyzed using a repeated measure ANOVA model or using a log-rank test.

Currently, GRPs transplantation in ALS is a promising therapeutic approach and clinical trials are in preparation (ClinicalTrials.gov Identifier: NCT02478450). We hypothesized that mGRPs transplantation in combination with immune modulatory therapeutics will impact the graft function and therefore modulate the course of ALS^5,23^. To test this hypothesis we transplanted allogenic mGRPs into the cisterna magna using a well-defined murine ALS model of transgenic SOD1^G93A^ mice in combination with immunomodulatory drugs: sPIF, Tac, and CB (Fig. 1A). Surprisingly, the mGRPs transplantation alone did not result in increased mice survival, which is in contrast to previous reports^24^. The addition of immunomodulatory regimes improved mice survival but did not reach statistical significance, while sPIF seemed to be superior compared to other immune modulatory drugs (Fig. 1B compare sPIF to other groups). We therefore focused on functional outcomes next.

Functional outcomes such as the hanging wire test are an excellent tool to detect early motor impairment, which impart quality of life^3^. The mGRPs transplantation alone did not impact mice performance measured by hanging wire test (Fig. 1C compare mGRPs to Sham). The additional application of immune modulatory drugs resulted in a significant delay of early motor impairment in the sPIF group only (Fig. 1C compare sPIF to other groups). This difference was especially significant in the first 60 days (Fig. 1C red line at 0.6 threshold) suggesting that sPIF predominantly impacted the early phase of the injury^25^. We aimed to investigate the grafts` survival and inflammatory responses in the brain next.

### Immunomodulatory therapeutics reduce peripheral immune response but not graft survival

**Figure 2:**
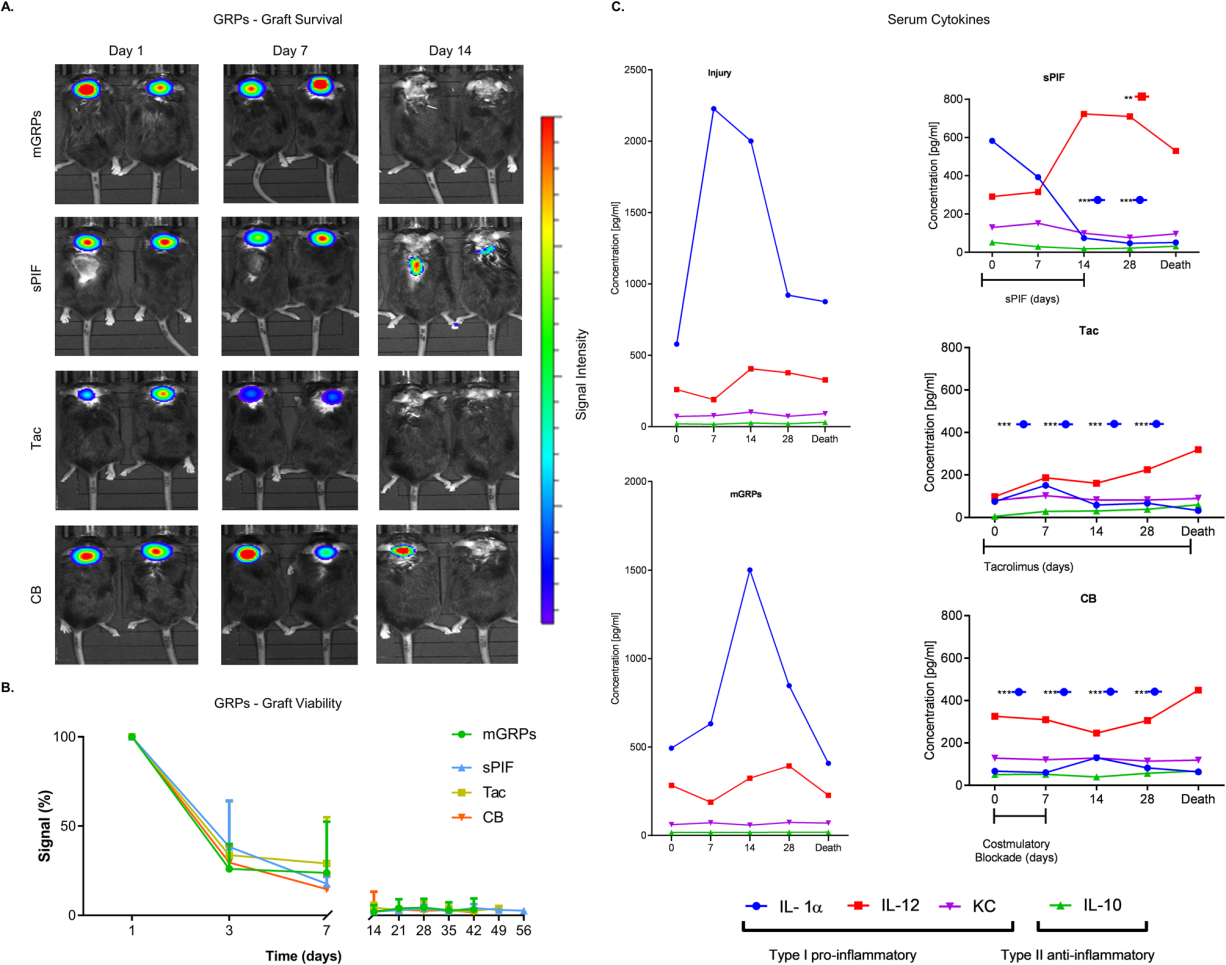
Graft survival and peripheral immune responses. (**A**) Representative bioluminescent images of the transplanted mGRPs and (**B**) graft survival over time. The immune modulators didn`t impact the graft survival. (**C**) Peripheral cytokines measured over time by groups with expected reduction of pro-inflammatory cytokine levels by additional use of immune modulators. mGRPs: glial-restricted precursors; sPIF: synthetic PreImplantation Factor; Tac: Tacrolimus, CB: co-stimulatory blockade. *p < 0.05; **p < 0.01; ***p < 0.001. Data are presented as mean ± SD (one-way analysis of variance (ANOVA) followed by a post–hoc test (Bonferroni test).

We measured the graft survival using Bioluminescent imaging first. We detected a rapid decline of graft viability during the first days with only marginal signal after day 14 (Fig. 2A and B compare mGRPs alone to other groups). This observation is in line with the lack of mGRPs` effect (Fig. 1). As the application of immune modulators did not impart the grafts` survival (Fig. 2A and B), we focused on immune responses next. Notably, GRPs modulate immune responses and result in neuroprotection^24^. As shown in Fig. 2C the SOD1^G93A^ transgenic mice showed expectantly increased peripheral pro-inflammatory IL-1α and IL-12 cytokines. The transplantation of mGRPs resulted in lower cytokines peaks but only with the additional application of immune modulators (Fig. 2C; compare sPIF, Tac and CB to Injury and mGRPs groups) which decreased IL-1α peak significantly. Interestingly, in the sPIF group IL-12 plateaued at day 28 (Fig. 2C; compare sPIF to other groups), which may be due to the different immune regimes. We reported the detailed effect of mGRPs on cytokines production recently^26^. We turned our attention towards the central nervous system next.

### mGRPs + sPIF reduce inflammation and preserve neuronal cells

**Figure 3:**
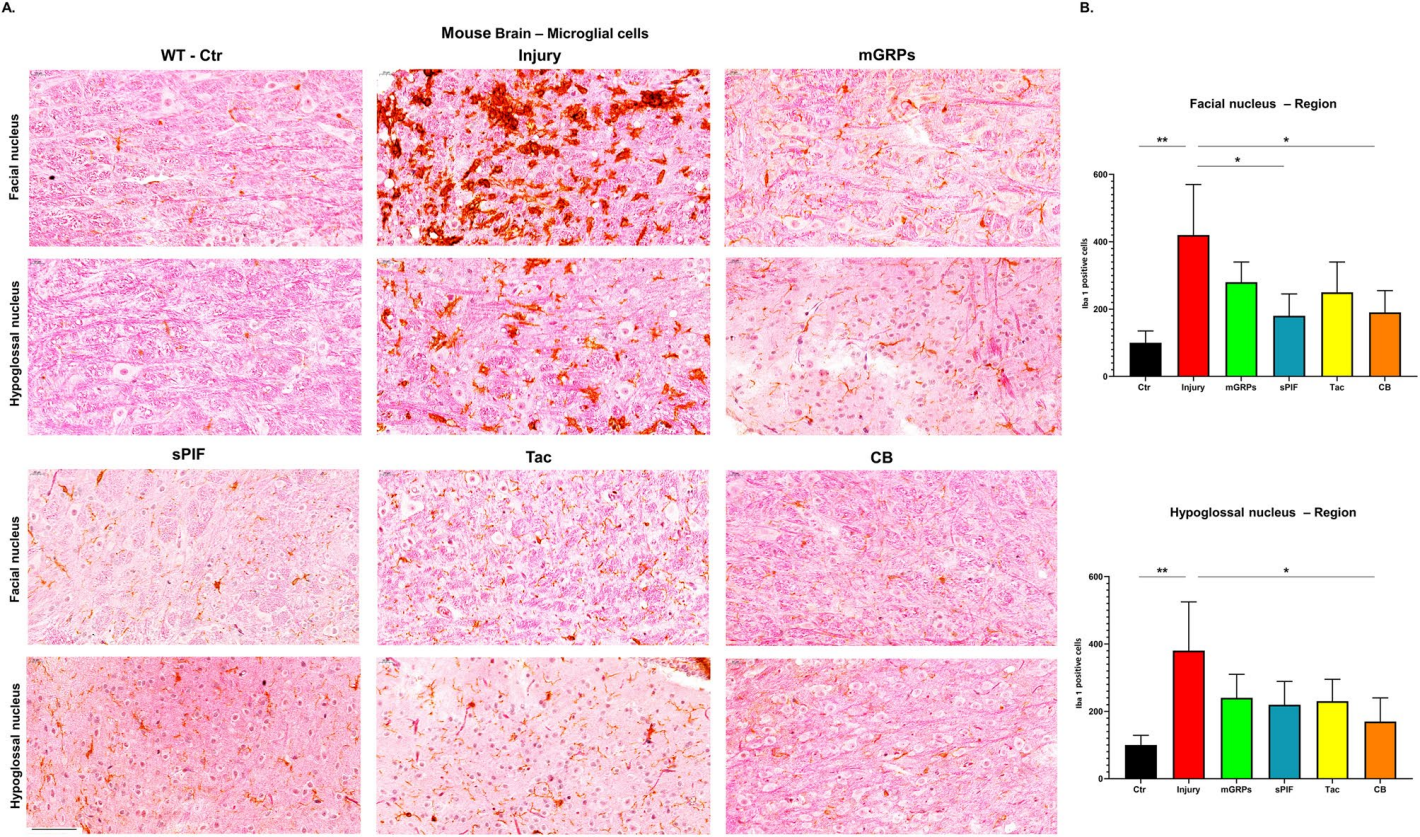
Central immune responses. (**A**) Representative images of microglial cells (Iba-1 positive cells) in two regions of the brain. (**B**) All therapeutic groups reduced the number of microglial cells significantly. mGRPs: glial-restricted precursors; sPIF: synthetic PreImplantation Factor; Tac: Tacrolimus, CB: co-stimulatory blockade. *p < 0.05; **p < 0.01; ***p < 0.001. Differences are calculated using one-way analysis of variance (ANOVA) followed by a post–hoc test (Bonferroni test).

**Figure 4:**
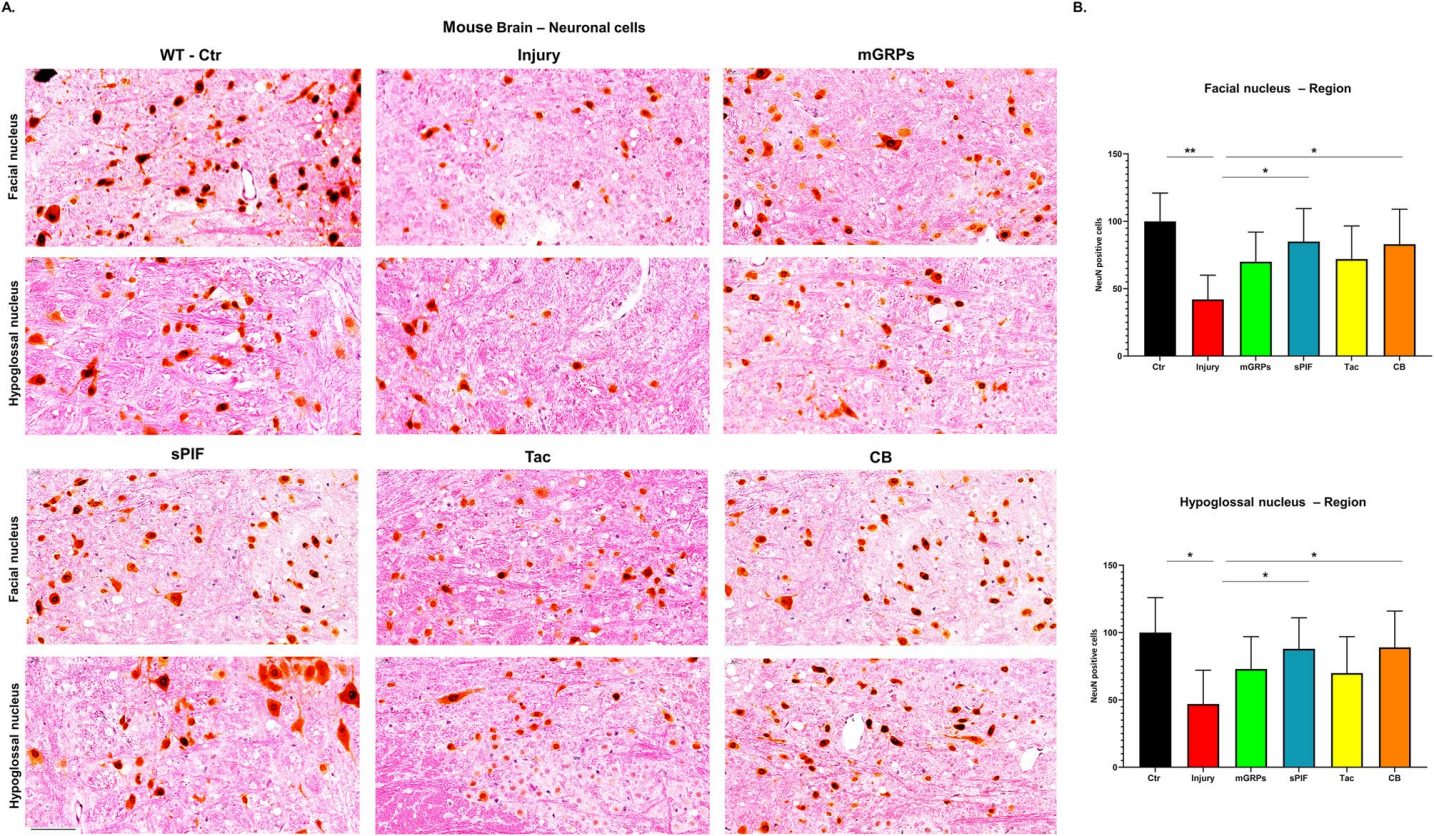
Neuronal survival. (**A**) Representative images of mature neurons cells (NeuN positive cells) in two regions of the brain. (**B**) All therapeutic groups reduced the number of microglial cells significantly. mGRPs: glial-restricted precursors; sPIF: synthetic PreImplantation Factor; Tac: Tacrolimus, CB: co-stimulatory blockade. *p < 0.05; **p < 0.01; ***p < 0.001. Differences are calculated using one-way analysis of variance (ANOVA) followed by a post-hoc test (Bonferroni test).

Given the functional improvements and peripheral modulation of cytokines by additional application of sPIF, Tac, and CB (Figs. 1C and 2C), we aimed to evaluate changes in the central nervous system next. We focused on Iba1 positive cells, as these are marker of microglial activation in the brain^12,27,28^. Expectantly, we detected increased number of Iba1 + cell in both motor nuclei regions (Fig. 3A and B; compare Ctr to Injury). The transplantation of mGRPs reduced the number of Iba1 + cells but the addition of sPIF or CB resulted in significant prevention of neuroinflammation (Fig. 3A and B; compare Injury to sPIF or CB). Notably, these results are in line with previous sPIF`s immune modulatory effect on the brain^12,28^. We evaluated the neuronal cells next and detected a reduced number of mature neurons in both motor nuclei regions (Fig. 4A and B compare Ctr to Injury). The transplantation of mGRPs restored the loss partially and again the application of both sPIF and CB resulted in significant protection of neuronal cells (Fig. 4A and B; compare Injury to sPIF or CB). These results are in line with both the modulation of peripheral (Fig. 2) and central (Fig. 3) immune responses, which we postulate to be responsible for the functional improvements (Fig. 1C).

### mGRPs + sPIF impart mGRPs progeny

**Figure 5:**
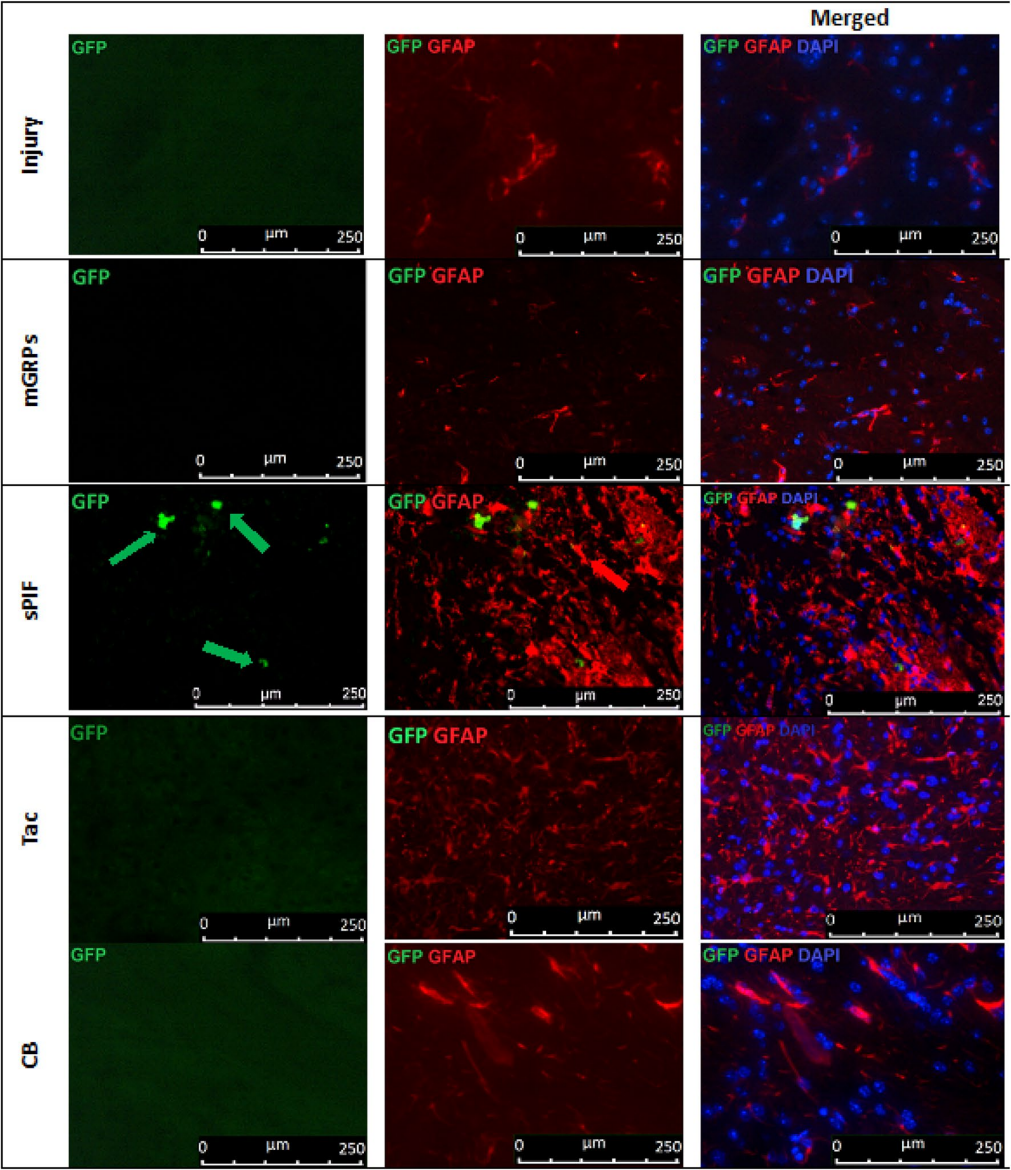
mGRPs progeny in brain parenchyma. Representative immunofluorescence images of astrocytes (GFAP + red marker) and mGRPs (GFP + green marker) in the diencephalon on day 56. Green arrows indicate positive cells in the sPIF group. mGRPs: glial-restricted precursors; sPIF: synthetic PreImplantation Factor; Tac: Tacrolimus, CB: co-stimulatory blockade.

**Figure 6:**
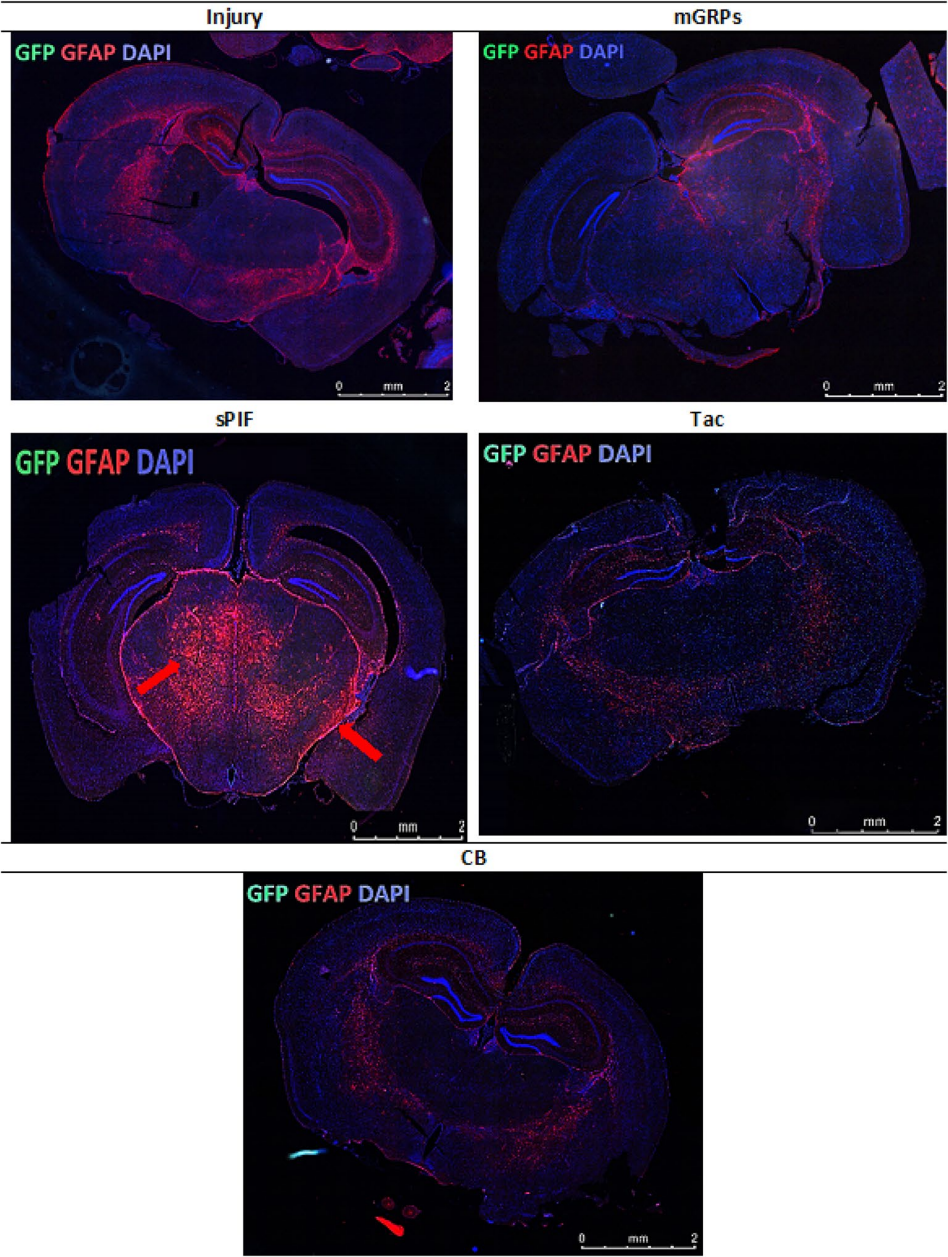
Immunofluorescence staining of mGRP cells (GFP + green marker) and astrocytes (GFAP + red marker) on whole cross section of the mouse diencephalon in the study groups. There can be seen a significantly larger area of astrocytes in the group of mice with sPIF administration (indicated by red arrows). mGRPs: glial-restricted precursors; sPIF: synthetic PreImplantation Factor; Tac: Tacrolimus, CB: co-stimulatory blockade.

**Figure 7:**
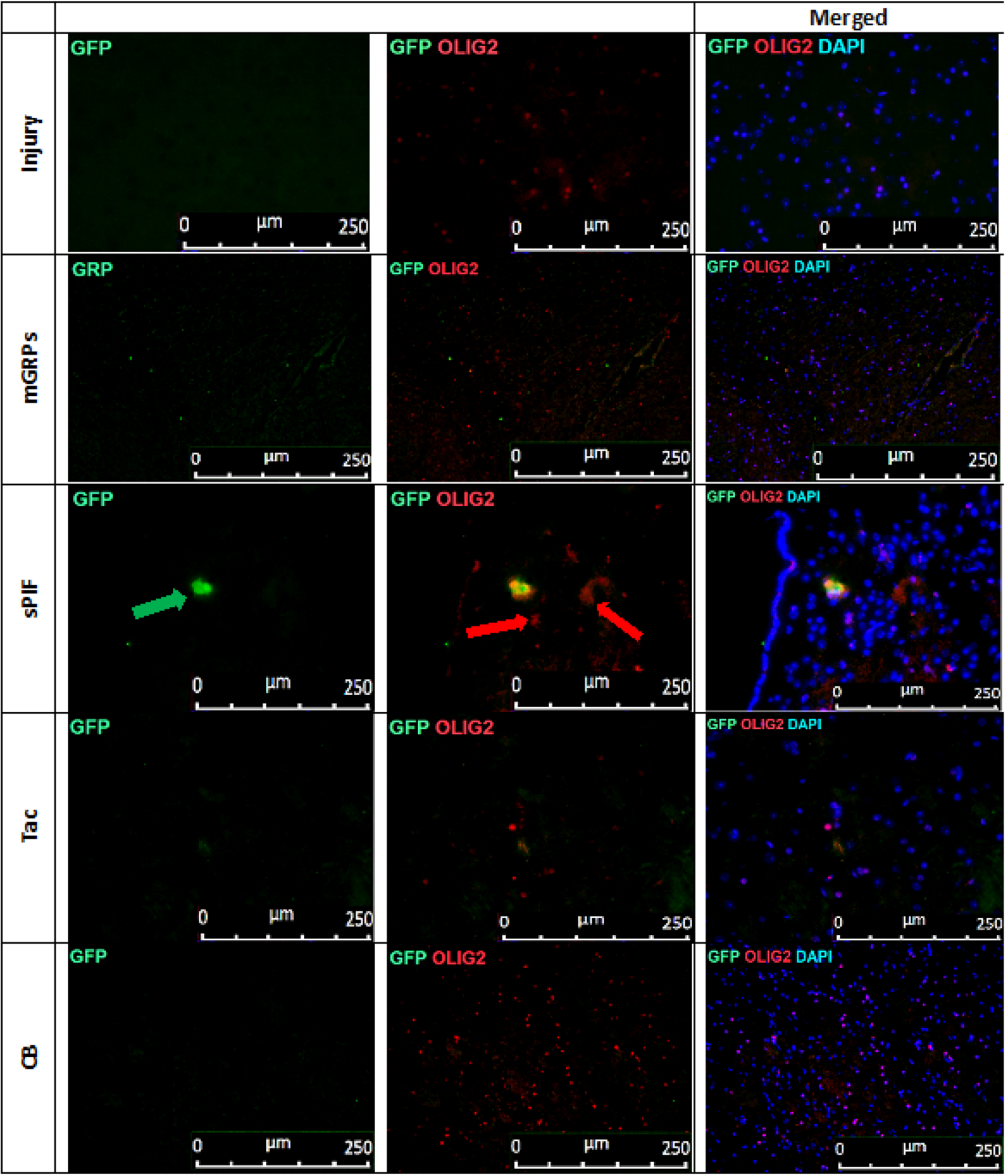
mGRPs progeny in brain parenchyma: representative immunofluorescence images of oligodendrocytes (OLIG2 + red marker) and mGRPs (GFP + green marker) in the diencephalon on day 56. Green arrows indicate positive cells in the sPIF group. mGRPs: glial-restricted precursors; sPIF: synthetic preimplantation factor; Tac: Tacrolimus, CB: co-stimulatory blockade.

**Figure 8:**
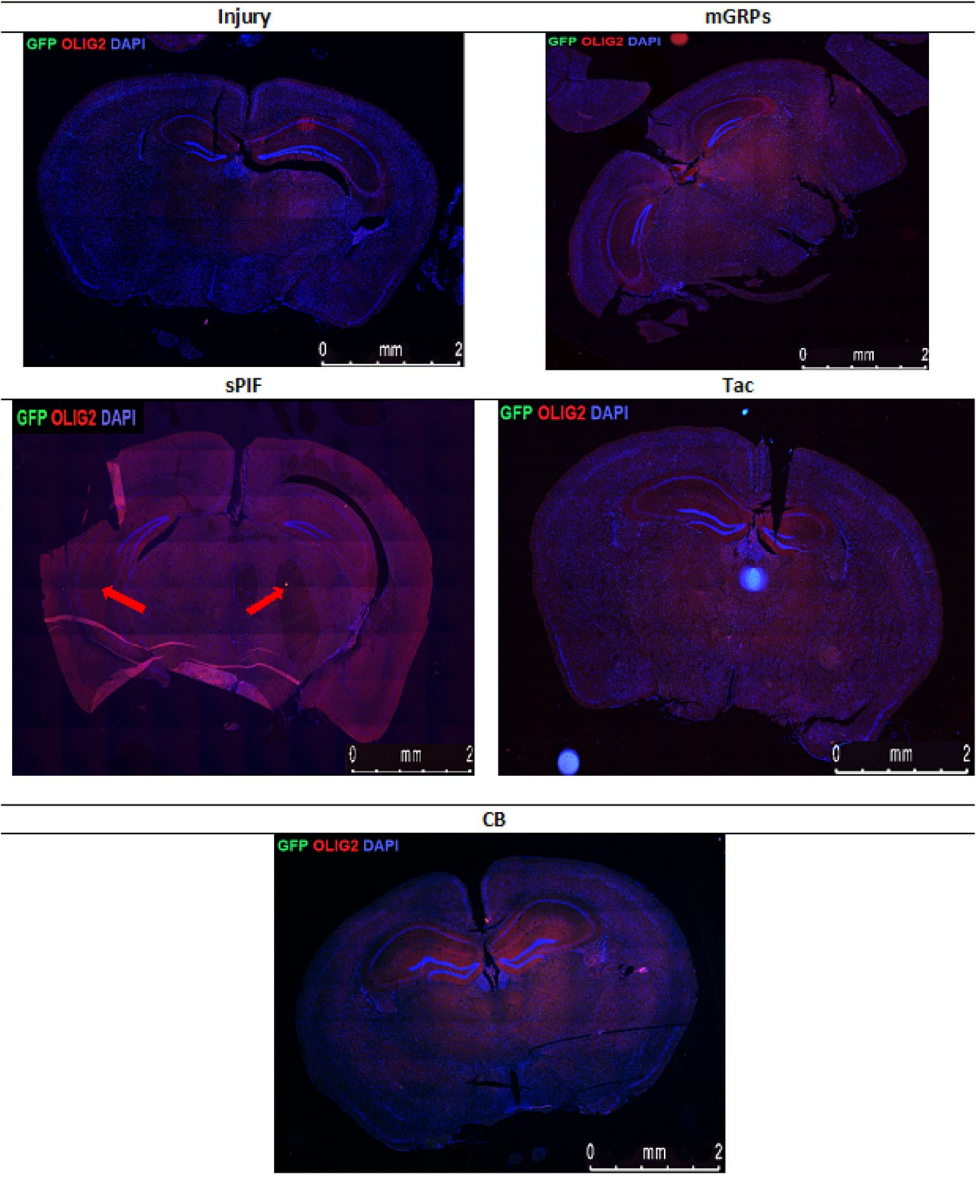
Immunofluorescence staining of mGRP cells (GFP + green marker) and oligodendrocytes (OLIG2 + red marker) on whole cross section of the mouse diencephalon in the study groups. There can be seen a larger area of oligodendrocytes in the group with sPIF administration (indicated by red arrows). mGRPs: glial-restricted precursors; sPIF: synthetic PreImplantation Factor; Tac: Tacrolimus, CB: co-stimulatory blockade.

We detected a functional improvement in mice despite the rapid decline of graft viability during the first days (Fig. 1C, Fig. 2A, B) compare mGRPs alone to other groups). Therefore, we decided to detect mGRPs progeny in the brains next^26^. Notably, glial precursors differentiate into astrocytes and oligodendrocytes and sPIF may affect their differentiation capacity^17,29,30^. Transplantation of mGRPs-^GFP+^ cells in the ALS transgenic mouse model (SOD1^G93A^) resulted in few GFP + astrocytes and oligodendrocytes in the brain parenchyma and this was evident in the sPIF group only (Figs. 5, 6, 7 and 8 compare mGRPs group to other groups). This observation is in line with the detected functional improvement (Fig. 1C compare sPIF to other groups) but whether sPIF administration alone and/or in combination with mGRPs transplant is beneficial warrant further investigation.

## Discussion

The transplantation of mGRPs alone (using our therapeutic regime) didn`t result in extended mice survival or functional improvement (Fig. 1). However, we here provide the first evidence that the additional use of immune modulatory drugs in ALS and especially sPIF can be promising. The combination of mGRPs and sPIF modulated not only the peripheral and central immune responses (Figs. 2 and 3) or neuronal survival (Fig. 4) but importantly improved functional outcomes as well (Fig. 1C). Given the lack of clear effect on graft viability (Fig. 2A and B) and its progeny (Figs. 5, 6, 7 and 8), the role of sPIF (alone or synergistic with mGRPs) is currently unclear. However, the neuroprotective effects of sPIF in both neonatal and adult murine models of brain injury open the possibility that sPIF alone may be neuroprotective in the ALS model as well^12,13,16,17^. The second successful immune modulatory drug was CB. Although CB is promising, the co-stimulatory molecules are expressed prominently in mice and therefore especially mice are vulnerable to immunological co-stimulatory blockade^31^. The use in humans is therefore not possible. Besides the modulation of innate immunity, the question of allogeneic versus semi-allogeneic transplantation and the principal role of GRPs implantation and potential histocompatibility discordance has to be considered^26^. In such case, sPIF, as a pregnancy derived peptide, may have an advantage and further ameliorate an autoimmune syndrome.

The lack of neuroprotection after mGPRs` transplantation without immune modulatory drugs is unclear. The transplantation regime including optimal cell graft source, phenotype, dose, and method of transplantation need further investigations^6^. We have to consider that GRPs source and phenotype differ depending on the species^31^ and the therapeutic agents need to be delivered and distributed broadly. The change of transplantation technique (for example use of MRI) or site (for example lumbar puncture) in combination with hydrogel-embedded vehicle may be beneficial^32,33^. The increase of cell number or viability to support cell migration to distant brain areas or the co-grafting of mesenchymal stem cells or its microvesicles may further increase the efficiency^34,35^. Importantly, however, GRPs injected to CNS and analyzed in post mortem tissues indicated pro-astrocyte (Fig. 6) and pro-oligodendrocyte (Fig. 8) differentiation exclusively in sPIF regime. Finally, preconditioning of mGRPs prior transplantation may affect the graft function as well.

We acknowledge that the chosen sPIF regime (starting day − 1 until day 14 after mGRPs transplantation) may not be sufficient. We speculate that longer sPIF treatment would have a more pronounced effect on long-term functional outcomes and perhaps on mice survival. We base this speculation on the early inflammation control (until day 14) and potential correlation with decreased functional performance afterwards (Fig. 1C). The longer application as in case of Tac or preconditioning of the cell graft can be envisioned as well. This longer (or even permanent) approach may maintain the GRPs transplant and/or suppress the autoimmune anti-SOD1 protein mutant reaction. Finally, we acknowledge that we didn’t take sex differences into account as the number of included animals was not sufficient and may impact the discordant results^24,25^. Further studies are ongoing but beyond the scope of this manuscript.

Altogether, the application of mGRPs imparts the course of ALS partially but the additional application of immune-modulatory therapeutics improved the neuroprotective effects significantly. The question whether sPIF alone would have been sufficient or if the mGRPs/sPIF would produce a synergistic effect needs further investigation. Given that sPIF is neuroprotective, crosses the blood–brain barrier, and already received a FAST-Track FDA approval (autoimmune diseases of non-pregnant subjects clinicaltrials.gov, NCT02239562), the clinical use could be very promising^12,13,17,28,36,37^.

## Materials and methods

### Animals and treatments

B6.Cg-Tg (SOD1^G93A^)1Gur/J (Animalab, The Jackson Laboratory, Bar Harbor, ME, USA, RRID: IMSR_JAX:004435) and SCID (Animalab, Charles River Laboratory, Wilmington, MA, USA, RRID: IMSR_JAX:001303) mice were obtained from JaxMice. SOD1^G93A^ animals were divided randomly into 5 groups and SCID animals were used as control. We defined animals (SOD1^G93A^ n = 6) with sham transplantation (surgery but no transplantation) as Injury, (SOD1^G93A^ n = 6) with mGRPs transplantation as (GRPs), (SOD1^G93A^ n = 6) with mGRP + sPIF as (sPIF), (SOD1^G93A^ n = 6) with mGRP + Tac as (Tac), and (SOD1^G93A^ n = 6) with mGRP + CB (MR1 + CTLA4) as (CB).

mGRP cells were received as a gift from the Department of Radiology and Radiological Science, Johns Hopkins School of Medicine (Baltimore, MD, USA). Cells were isolated as previously described from spinal cords dissected from Luc + /PLP/GFP + mice between E12.5 and E14 stage^35^. Briefly, cells were cultured in DMEM/F12 medium (Thermo Fisher, Waltham, MA, USA), supplemented with N2 and B27 (Life Technologies, Carlsbad, CA, USA), 1% BSA (Abcam, Cambridge, UK), Penicilin-streptomycin (Life Technologies, Carlsbad, CA, USA) and 20 ng per mL bFGF (Peprotech, Rocky Hill, NJ, USA). Cell culture flasks were covered with poly-L-lysine and laminin (Sigma, Saint Louis, MO, USA). The cells were cultured until monolayer was achieved, detached from culture flasks and characterized as previously described in terms of neural and glial cell markers, pro-neurogenic phenotype and their potential to differentiate into astrocytes^31^. mGRP cells were detached from the culture flask with TrypLE (Thermo Fisher, Waltham, MA, USA) enzymatic digestion. Reaction was disrupted by adding PBS in 1:3 proportion. Cells were centrifuged 5 min with 1000 RPM. The viability of the mGRPs was assessed with trypan blue exclusion and only suspensions with over 70% of cell viability were used for transplantation. The suspension was prepared out of 0.5 mln mGRP cells in 10 µl of PBS. Prior to surgery, we assured acclimatization of all animals to the laboratory environment and we provided animals with food and water ad libitum. We used controlled conditions of light (12 h light/12 h dark) and temperature (23–25 °C) for animal housing. We followed aseptic rodent survival surgery guidelines. Prior to cell transplantation mice were anesthetized with 5% (induction) and maintained in 2% isoflurane. Animals were immobilized in stereotaxic device (Leica, Wetzlar, Germany). 10 µl of cell suspension was administered into cisterna magna 2 µl/min via infusion pump (KD Scientific, Hollston, MA, USA).

We synthesized synthetic PIF_15_ (MVRIKPGSANKPSDD) by solid-phase peptide synthesis (Peptide Synthesizer, Applied Biosystems) employing Fmoc (9-fluorenylmethoxycarbonyl) chemistry at Bio-Synthesis, Inc. (Louisville, TX, USA) as previously published^38^. Briefly, we carried out final purification by reversed-phase HPLC and we verified the identity by matrix-assisted laser desorption/ionization time-of-flight mass spectrometry (amino acid analysis at > 95% purity). sPIF was applied in amount of 1 mg/kg body weight starting at day -1 prior to graft administration, and administered for 2 weeks after mGRPs transplantation (Fig. 1A).

Tacrolimus (Sigma Aldrich, St.Louis, MO, USA) was applied in amount of 1 mg/kg body weight starting at day − 1 prior to grafting and administered every day until the end of observation (day 56; Fig. 1A). Costimulatory Blockade (CB) included MR1 (CD40L, Hozel Diagnostika, Bio X Cell, Lebanon, NH, USA, RRID: AB_1107601) and CTLA4 (Orencia, Bristol-Myers Squibb Company, New York, NY, USA), which were administered at amount 25 mg/kg at days 0, 2, 4 and 6 (day 0 defined as day of transplantation; Fig. 1A).

The immune modulatory regimes were chosen based on previous reports of successful immune modulation and neuroprotective property^11,17,39–41^.

All used procedures followed the requirements of Commission Directive 86/609/EEC, which concern the protection of animals used for experimental and other scientific purposes. We acquired Local Ethical Committee for Animal Experimentation, Poznan University of Life Sciences approval on preclinical studies (No. 12/2017” Application of glial progenitors for treatment of ALS”). We should like also to confirm that all animal experiments were performed in accordance with relevant guidelines and regulations. Additionally, the reporting in the manuscript follows the recommendations in the ARRIVE guidelines.

### Serum collection and cytokine analysis

We collected samples every week, starting from day of graft transplantation (day 0), 100 µl of blood from mouse tail. Blood was stored 1 h in room temperature, and overnight in + 4 °C. Serum was gently aspirated, and centrifuged 10 min with 2000 RPM to separate blood morphotic bodies. Purified serum was stored in − 80 °C until further analysis. Multiplex ELISA 23-cytokine kit (M60009RDPD) was obtained from Bio-Rad (Bio-Plex Pro Mouse Cytokine 23-plex Assay #M60009RDPD, Hercules, Bio-Rad, CA, USA, RRID: AB_2857368) and performed according to instructions. 50 µl of diluted in sample diluent, 1:4 serum samples and diluted at range 1:10–1:20. Measurement standards were used, diluted 9 times according to the protocol and added at template plate. Vortexed beads were added to the assay 96-well plate. Plate was again washed 3 times with wash buffer, then from template plate, 50 µl of samples, standards and blank were transferred to measurement plate with multichannel pipette. Plate was sealed with tape and incubated 30 min. After this time, plate was washed 3 times. 25 µl of detection antibody was added to the wells with multichannel pipette. Plate was sealed and incubated for 30 min. and next washed 3 times using wash buffer. 50 µl per well Streptavidin-PE was added, with multichannel pipette. The plate was sealed, and incubated for 10 min. Wells were washed 3 times with wash buffer. Beads were resuspended with 125 µl of assay buffer and analysis was performed with Bio Plex 200 reader.

### Functional testing and bioluminescent imaging (BLI) of graft survival

To assess the motor capacity of our treatment groups, we performed motor skills assays^3^. Starting at day -7 SOD1^G93A^ mice (n = 30) were trained in hanging wire. Animals were trained 3 times a week in 2 days interval. We performed weekly measurement starting at day 0 until the end of the experiment. Hanging wire test was used to test neuromuscular strength and coordination^3^. Briefly, mice were hung on a wire and had to catch the wire both by upper and lower limbs as well as tangle it with tail in order to move the whole distance towards a stick (repeated three times for each mouse and obtaining an average). Maximum points (1.5 point) were given when mice performed the task. Medium score (1 point) were given when mice were able to accomplish the whole task, but catching wire with both limbs and tail took more than 5 s. Low score (0.66 point) was given when mouse was able to accomplish the task only using upper limbs. Poor score (< 0.66 points) was given when mouse was unable to move the whole distance and we counted the seconds, which mice were able to hang on the wire (1 s = 0.016 points). All functional testing was performed in a blinded manner.

We performed imaging at days 1, 3, 7, and then weekly. We performed measurements using IVIS Lumina IT series III (Perkin Elmer, Waltham, Massachusetts, USA) and Xeno Light Luciferin (Perkin Elmer, Waltham, MA, USA) 150 mg per 1 kg. We defined time optimal enzyme activity at 10 min.

### Tissue collection and immunohistochemical staining

At the end of experiment, mice were subjected to full body perfusion with 20% sucrose and 10% buffered formalin suitable for histology assays (Sigma Aldrich, St.Louis, MO, USA). Mice were decapitated and incubated in formalin for 24 h. Following the tissues (brain and spinal cord) were isolated, paraffin fixed for further analyses. After brain removal we fixed the tissue in formaldehyde solution (4%) for 2–4 h at RT followed by 4 °C for a total time of 24–48 h. Fixed tissues were embedded in paraffin and finally sectioned into 7 µm slices. After deparaffinization of the slides (xylol 3 × 3 min, EtOH 100% 2 × 3 min, EtOH 95% 1 × 3 min, EtOH 70% 1 × 3 min and dest. H_2_O), the target was retrieved in Citrate Buffer (10 mM, pH = 6.0) in a pressure cooker for 12 min and then they were let cool down for another 20 min. Slides were washed in 0.1% Tween-20/TBS (pH7.6) and blocked 1 h in 10% goat serum/1% bovine albumin/1%TritonX in TBS (pH7.6). One part of the slides was incubated overnight with a rabbit monoclonal Neuronal antibody (NeuN, Abcam Cat# ab177487, RRID:AB_2532109) at 1:5000. Other slides were washed in 0.1% Tween-20/TBS (pH7.6) and blocked one hour in 10% rabbit serum/1% bovine albumin/1%TritonX in TBS (pH7.6). Iba1 antibody (Abcam Cat# ab5076, RRID:AB_2224402) at 1:100 concentration was applied on these slides during 24 h. Following first antibody incubation, slides were washed in 0.1% Tween-20/TBS (3 × 5 min) and incubated in endogenous peroxidase blocking solution at RT for 15 min. Peroxidase-labeled polymer (DAKO anti rabbit or anti goat) was applied to the slides for one hour at RT. Slides were washed in in 0.1% Tween-20/TBS (3 × 5 min), followed by application of DAB + chromogen in buffer substrate for 10–30 min, according to the manufacturer’s instructions (DAKO EnVision + System-HRP (DAB), K4007). Slides were rinsed in ddH_2_O, counterstained in hematoxylin–eosin and dehydrated in a series of ethanol baths (95 > 100%) and xylene, and mounted with Eukitt (Medite Service AG).

Assessment of neuronal and microglial positive cells were performed in the region of interest (ROI). We defined ROI as the brainstem (facial and hypoglossal) motor nuclei as these were reported to be affected in SOD1^G93A^ model previously^42^. All images of immunohistochemical stainings were obtained with a Zeiss based microscope (Pannoramic, 3CCD camera Hitachi HV-F22CL 1,4MP and Zeiss AxioCam MRm monochrome camera) equipped with a digital camera. We used 40 × or 60 × objective to acquire images for ROI evaluation. Consecutive coronal sections per animal and for each specific immunostaining were acquired by an independent observer blinded to the experimental conditions. We analyzed and reconstructed the images using Image J (US National Institutes of Health).

### Immunofluorescence staining of the brain tissues

Paraffin slides of the brain tissue after heated in 1 h in 60 °C were deparaffinized in: 1 × 10 min. Xylen I, 1 × 10 min Xylen II, 1 × 10 min EtOH 100%,1 × 10 min EtOH 70%,1 × 10 min EtOH 40%, 1 × 10 min dest.H_2_O. The target was retrieved in Citrate Buffer + Tween 20(10 mM, pH = 6.0) in a water bath for 20 min in 97 °C and then they were let cool down for another 20 min. Slides were washed in PBS three times and blocked 1 h in 10% Goat serum + 1% bovine albumin serum. All slides were incubated overnight at 4 °C with a first primary mouse Anti-GFP antibody 1:500 (Abcam Cat# ab1218, RRID:AB_298911). Washed three times with PBS, then secondary antibody Goat Anti-Mouse IgG H&L (Alexa Fluor® 488) in concentration 1:700 (Abcam Cat# ab150117, RRID:AB_2688012) was applied for 40 min in RT. Then one part of the slides was washed three times in PBS and the second primary Rabbit Anti-GFAP antibody (Abcam Cat# ab7260, RRID:AB_305808) was applied 1:1000, incubated overnight at 4 °C. After that the secondary antibody Goat Anti-Rabbit IgG H&L (Alexa Fluor® 594) (Abcam Cat# ab150080, RRID:AB_2650602) was applied for 40 min RT in concentration 1:700. The second part of the slides was incubated with Rabbit Recombinant Anti-Olig2 antibody (Abcam Cat# ab109186, RRID:AB_10861310) 1:100 incubated overnight in 4 °C. Next day the secondary antibody Goat Anti-Rabbit IgG H&L (Alexa Fluor® 594) (Abcam Cat# ab150080, RRID:AB_2650602) was applied for 40 min RT in 1:700 concentration. All slides were washed three times in PBS and cell nuclei were stained with DAPI (VECTASHIELD® Antifade Mounting Medium with DAPI, Vector Laboratories, Burlingame, CA, USA, Cat# H-1200, RRID: AB_2336790) for 30 min in RT. The slides were washed in PBS, mounted with DAKO mounting medium (Agilent Technologies, Santa Clara, CA, United States) and analyzed under Leica DM5500 fluorescent microscopy with LAS X software.

### Quantification and statistical analysis

We performed all quantifications including the manual cell counts in a blinded manner. We counted NeuN and Iba1 positive cells in the ROI (see above) as previously reported^43–45^. We determined the number of cells by unbiased counting of positive cells^43^. We presented the results as the mean ± standard deviation (mean ± SD). We used one-way analysis of variance (ANOVA) followed by a post–hoc test (Bonferroni test) for IHC and ELISA Analyses. Considering the effect of therapy on physical activity across time we first described the temporal variation of the physical activity of each mouse group by calculating, at each time point and for each group the mean physical activity and the associated 95%-confidence interval. For the 5 groups, we then investigated the effect of the therapy on the physical ability of the mice. Differences between groups were analysed using a repeated measure ANOVA model with a fixed effect for the group, of the time and a random intercept for the mouse. In a second, more explorative, approach we determined, for each group the time when the physical activity was first measured below a certain threshold. This “time to reduced activity” was then compared between groups using an ANOVA model with a fixed effect for group. The clinically relevant threshold was determined according to previous observations and defined as value 0.9 for the Wire Test. Finally, to assess difference in survival between groups, we used Kaplan–Meier curves by group. Differences between survival curves were tested using a log-rank test. We determined statistical significance at *p* < 0.05.
